# Heterologous Expression of the Wheat Aquaporin Gene *TaTIP2;2* Compromises the Abiotic Stress Tolerance of *Arabidopsis thaliana*

**DOI:** 10.1371/journal.pone.0079618

**Published:** 2013-11-04

**Authors:** Chunhui Xu, Meng Wang, Li Zhou, Taiyong Quan, Guangmin Xia

**Affiliations:** The Key Laboratory of Plant Cell Engineering and Germplasm Innovation, Ministry of Education, School of Life Sciences, Shandong University, Jinan, Shandong, China; International Rice Research Institute, Philippines

## Abstract

Aquaporins are channel proteins which transport water across cell membranes. We show that the bread wheat aquaporin gene *TaTIP2;2* maps to the long arm of chromosome 7b and that its product localizes to the endomembrane system. The gene is expressed constitutively in both the root and the leaf, and is down-regulated by salinity and drought stress. Salinity stress induced an increased level of C-methylation within the CNG trinucleotides in the *TaTIP2;2* promoter region. The heterologous expression of *TaTIP2;2* in *Arabidopsis thaliana* compromised its drought and salinity tolerance, suggesting that *TaTIP2;2* may be a negative regulator of abiotic stress. The proline content of transgenic *A. thaliana* plants fell, consistent with the down-regulation of *P5CS1*, while the expression of *SOS1*, *SOS2*, *SOS3*, *CBF3* and *DREB2A*, which are all stress tolerance-related genes acting in an ABA-independent fashion, was also down-regulated. The supply of exogenous ABA had little effect either on *TaTIP2;2* expression in wheat or on the phenotype of transgenic *A. thaliana*. The expression level of the ABA signalling genes *ABI1*, *ABI2* and *ABF3* remained unaltered in the transgenic *A. thaliana* plants. Thus *TaTIP2;2* probably regulates the response to stress via an ABA-independent pathway(s).

## Introduction

A range of abiotic stresses, including soil salinity, drought and extreme temperature, can compromise crop yield and quality. Improving tolerance to these stresses is thus a major priority in many crop breeding programmes. The effectiveness of water transporters, such as the aquaporins, is an important component of the plant response to stress [1]. The aquaporins belong to a highly conserved major intrinsic protein family, and combine with the cell membrane system to control the flow of water between and within the cell. Their structure is characterized by the formation of six transmembrane domains connected by five loops [2–4]. Based on their sub-cellular localization and sequence, plant aquaporins have been classified into four sub-families, namely the plasma membrane intrinsic proteins (PIPs), the tonoplast intrinsic proteins (TIPs), the nodulin 26-like intrinsic proteins (NIPs) and the small basic intrinsic proteins [5]. There are also three new subclasses of aquaporins in moss *Physcomitrella patens*, named GlpF-like intrinsic protein (GIP), hybrid intrinsic protein (HIP) and X intrinsic protein (XIP) [6].

The *Panax ginseng* aquaporin gene *PgTIP1* [7], tomato (*Solanum lycopersicum*) *SlTIP2;2* [8] and cotton (*Gossypium hirsutum*) *GhPIP2;7* [9] have been shown to positively regulate salinity and drought tolerance, but negative effects on stress tolerance of certain aquaporins have also been documented [10]. The stress inducibility of aquaporin genes is variable, with examples including both their up- [11–15] and down-regulation [13,14,16,17] as well as their insensitivity [13–15]. Among the wheat aquaporins, it is known that certain TIPs show a marked affinity with ammonia [18], while the activity of particular *NIP* [19] and *PIP* genes [20–22] have been associated with an improved response to abiotic stress. Of a set of 35 wheat aquaporin genes, 24 were shown to be *PIP*s and 11 were *TIP*s that have diverse sequence characteristics [23]. However, as yet there is an insufficient understanding of the role of the aquaporins in the abiotic stress response of wheat.

The bread wheat cultivar Shanrong No. 3 (SR3) is a derivative of an asymmetric somatic hybrid between cv. Jinan 177 (JN177) and an accession of tall wheatgrass (*Thinopyrum ponticum*) [24]. SR3 has proven to show an enhanced level of both salinity and drought tolerance over JN177 [25]. A microarray-based gene expression study has shown that in drought and salinity stressed SR3 plants, *TaTIP2* was down-regulated [26]. Here, we report the isolation of the *TaTIP2;2* SR3 allele. We have determined its chromosomal location and confirmed that its expression is suppressed by both drought and salinity stress. We show that it encodes a protein deposited in the endomembrane, and that its heterologous expression in *A. thaliana* compromised the level of tolerance to salinity and drought stress. Finally, we demonstrate that the gene is involved in the down-regulation of proline synthesis and acts in an ABA-independent manner.

## Materials and Methods

### Plant materials and growing conditions

SR3 seedlings were raised in half-strength Hoagland's liquid medium [27] at 22°C under a 16h photoperiod with the light intensity of 3,000 lux. At the three leaf stage, a portion of the seedlings was exposed to abiotic stress by the addition to the medium of either 150mM NaCl, 18% w/v PEG or 100μM ABA. After 0h, 0.5h or 48h of this treatment, RNA was extracted from both the leaf and the root using an RNAiso plus kit (Takara, Dalian, China), following the protocol recommended by the manufacturer. *A. thaliana* plants were cultured on either half strength MS medium or soil under the same environmental conditions as the wheat seedlings.

### Isolation of *TaTIP2;2*

The *TaTIP2;2* cDNA sequence (GenBank accession number AY525640) was used to design a primer pair (TaTIP2, sequences given in Table 1) able to amplify the gene's open reading frame from a template of cDNA prepared from salinity stressed SR3 seedlings. For cloning purposes, an *Xba*I restriction site was included in the forward primer and a *Sac*I site in the reverse primer. The PCR comprised an initial denaturation of 95°C/3min, followed by 35 cycles of 94°C/40s, 65°C/50s, 72°C/60s, ending with a final extension of 72°C/10min. The amplicons were gel-purified, digested with *Xba*I and *Sac*I and ligated with *Xba*I/*Sac*I digested pSTART [28]. The resulting construct was transferred into *Agrobacterium tumefaciens* strain EHA105 for the agroinfection of *A. thaliana* Col-0 via the floral dip method [29]. Homozygous transgenic segregants in the T_3_ generation were used for phenotypic and gene expression analysis. The same primer pair was used to recover the SR3 *TaTIP2;2* genomic sequence, and the resulting amplicon was gel-purified and ligated with the pMD18-T vector (Takara, Dalian, China) to allow for its sequencing.

**Table 1:** Sequences of PCR primers used.

Name	Forward sequence (5’-3’)	Reverse sequence (5’-3’)
TaTIP2	GCTCTAGAATGCCGGGCTCCATCGCCTTCG	CGAGCTCTTAGTAGTCGTTGCCGGCGACGGA
TaTIP2;2	CTCTCATCCTCCCAGTTCTGTTC	CACGTACCGGTAGACGACGC
Actin	GTTCCAATCTATGAGGGATACACGC	GAACCTCCACTGAGAACAACATTACC
GC island1	TTGGTGGTTATATAATTTTGGAGGT	CAAAACAATTTTTCAAATCCAATAC
GC island2	TGTTAAGGGGGAAGTTGATATTTA	AAAAAATACCATAACATACACCAAC
TaTIP2;2-GFP	GCTCTAGAATGCCGGGCTCCATCGCCTTCG	GCGGATCCGTAGTCGTTGCCGGCGACGGA

A transmembrane domain prediction of the predicted TaTIP2;2 protein was obtained using the TMHMM tool provided at http://www.cbs.dtu.dk/services/TMHMM. The software package DNAMAN v6.0 (http://www.lynnon.com/) was employed to obtain the intron/exon structure of the genomic sequence. The phylogeny of TaTIP2;2 was investigated by comparing its protein sequence with those of TIP sequences from *A. thaliana*, rice, maize, barley and wheat represented in the GenBank database.

### Chromosomal location of *TaTIP2;2*

The genomic DNA of a full set of wheat cv. Chinese Spring nulli-tetrasomic lines [30] and a partial set of ditelocentric lines [31] was used as a template for PCRs primed with TaTIP2. The PCR comprised an initial denaturation of 95°C/5min, followed by 35 cycles of 94°C/30s, 65°C/40s, 72°C/60s, ending with a final extension of 72°C/10min. The resulting amplicons were separated by agarose electrophoresis.

### Analysis of *TaTIP2;2* expression

The cDNA first strand was synthesized using a Tianscript RT kit (Tiangen, Beijing, China), and this was used as the template for a semi-quantitative RT-PCR (sqRT-PCR), primed by TaTIP2;2 (primer sequences given in Table 1). The wheat *Actin* gene (GenBank accession AB181991) was used as a reference (primer sequences given in Table 1). The PCR comprised an initial denaturation of 95°C/5min, followed by 25-30 cycles of 94°C/30s, 55°C/30s, 72°C/60s, ending with a final extension of 72°C/10min.

### Methylation analysis of *TaTIP2;2* promoter

The methylation status of the *TaTIP2;2* promoter was investigated both before and after the imposition of salinity stress (150mM NaCl for 48h). The 2.5kbp sequence upstream of the *TaTIP2;2* start codon was obtained from a draft assembly of the wheat cv. Chinese Spring genomic sequence (http://www.cerealsdb.uk.net/CerealsDB/Documents/DOC_search_reads.php). Bisulphate sequencing was applied to genomic DNA extracted from SR3 at the three leaf stage. The DNA was processed with a sodium bisulphate kit (Qiagen, Hilden, Germany) and then used as a PCR template. The detection of CG islands and the design of primers were facilitated by MethPrimer software [32], and the resulting primer sequences are given in Table 1. The PCR comprised an initial denaturation of 94°C/4min, followed by 35 cycles of 94°C/30s, 60°C/30s, 72°C/30s, ending with a final extension of 72°C/10min. The amplified fragments were gel-purified, ligated with the pEASY T1 vector (Transgene) and transformed into *E. coli*. A random selection of at least 15 clones per amplicon was sequenced. BiQ Analyzer software (http://biq-analyzer.bioinf.mpi-inf.mpg.de/) was used to calculate the the ratios of cytosine methylation at each CG/CNG/CNN site. 

### Sub-cellular localization of *TaTIP2;2* in wheat protoplasts

The *TaTIP2;2* gene without its stop codon was cloned into the *Xba*I and *Bam*HI sites of the pUC-GFP vector to form a *TaTIP2;2::GFP* gene fusion. The sequence was amplified using the primer pair TaTIP2;2::GFP (primer sequences given in Table 1), and transferred into wheat cv. Yangmai158 protoplasts isolated from embryogenic calli to perform a transient expression analysis, following the methods described by Yoo et al. [33]. GFP fluorescence was monitored with a Leica TCS SP2 confocal laser scanning microscope under 488nm excitation.

### Phenotyping of transgenic *A. thaliana*

Seed of both wild type and transgenic *A. thaliana* was surface-sterilized (0.1% w/v HgCl_2_, 15min), rinsed five times in water, and held at 4°C on half strength MS medium for 36h. To investigate the response to drought and salinity stress, a portion of the seeds was then held at 22°C for 3d, following which they were transferred onto half strength MS medium containing either 300mM mannitol (drought treatment) or 150mM NaCl (salinity treatment). The plates were orientated vertically and held for 10d at 22°C. A second portion of the seed was used to measure seedling proline content. For this purpose, three week old seedlings cultured on MS medium were processed as described by Troll and Lindsley [34].

### The expression of stress-related genes in transgenic *A. thaliana*

RNA of four week old *A. thaliana* plants on half strength MS was extracted with the same method for SR3 and used as a template for quantitative RT-PCRs targeting the stress-related genes *ABI1*, *ABI2*, *ABF3*, *SOS1*, *SOS2*, *SOS3*, *CBF3*, *DREB2A*, *DREB2B*, *MYB2*, *MYC2*, *RAB18*, *RD29B* and *P5CS1*. Relevant primer sequences and the analytical method adopted were as reported elsewhere [35,36].

## Results

### The sequence characteristics of *TaTIP2;2*

**Figure 1:**
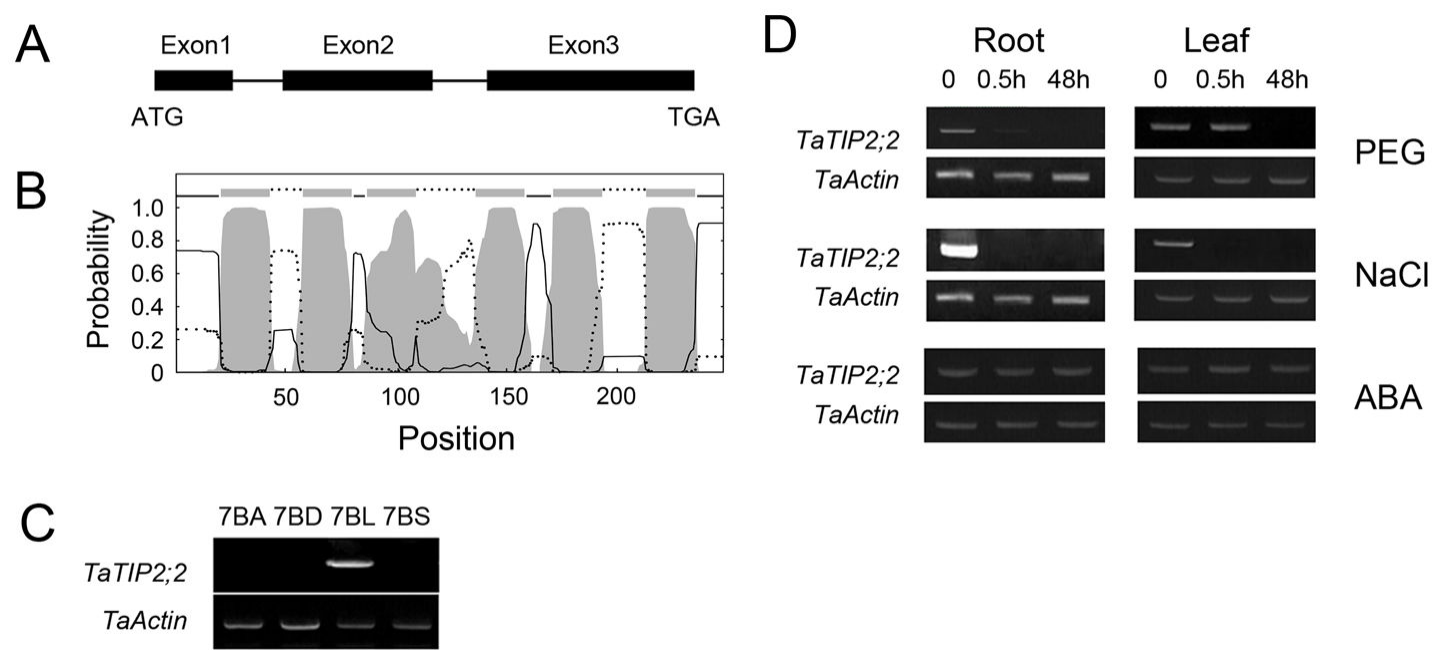
Characteristics of *TaTIP2;2*. (A) The genomic copy consists of three exons (indicated by filled bars) and two introns (lines). (B) Domain prediction of the gene product. Internal residues indicated by full lines, external ones by dotted lines. The transmembrane domains are shaded grey. (C) Chromosomal location of *TaTIP2;2* based on aneuploid stocks of cv. Chinese Spring. 7BA, 7BD are deficient for chromosome 7B, 7BS for chromosome arm 7BL and 7BL for chromosome arm 7BS. (D) sqRT-PCR analysis shows that *TaTIP2;2* was expressed in both the root and leaf, and was down-regulated by drought (PEG) and salinity, but not by ABA.

The SR3 *TaTIP2;2* ORF comprises a 747bp sequence, predicted to encode a 248 residue polypeptide. Its sequence is the same with the previously reported *TaTIP2;2* (GenBank accession number AY525640) [18]. Alignment of its opening reading frame with the *TaTIP2;2* genomic sequence showed that the genomic copy consists of three exons (130bp, 249bp and 367bp) and two introns (83bp and 92bp) (Figure 1A). TMHMM analysis indicated that the protein contains six transmembrane domains (Figure 1B), typical of the aquaporins. An alignment of TaTIP2;2 protein with other plant TIP proteins confirmed the conventional classification of the aquaporin gene family, the three wheat TIP2 proteins clustered closely with one another, and their sequence was ~89% homologous with that of the rice homologue OsTIP2;2 (Figure S1).

When DNA of the Chinese Spring nulli-tetrasomic lines was amplified with the TaTIP2 primer pair, only lines deficient for chromosome 7B (7BA and 7BD) failed to amplify the expected product. The PCR profile of the ditelocentric line carrying the long arm but not the short arm of 7B (7BL) was the same as that of the euploid, while that of the line carrying the short arm but not the long arm (7BS) was the same as that of the lines deficient for chromosome 7B (Figure 1C). Thus the gene must be located on the long arm of chromosome 7B.

### Expression of *TaTIP2;2* in response to abiotic stress and exogenous ABA

The outcome of the sqRT-PCR experiments showed that *TaTIP2;2* was expressed in both the leaf and root of SR3 (Figure 1D). In the root, the gene was down-regulated following a 0.5h exposure to either salinity or drought stress. In the leaf, it was also down-regulated following a 0.5h exposure to salinity, but its down-regulation in response to PEG treatment was delayed. There was no apparent effect of exogenous ABA on its expression (Figure 1D).

### Methylation of the *TaTIP2;2* promoter under stress treatment

**Figure 2:**
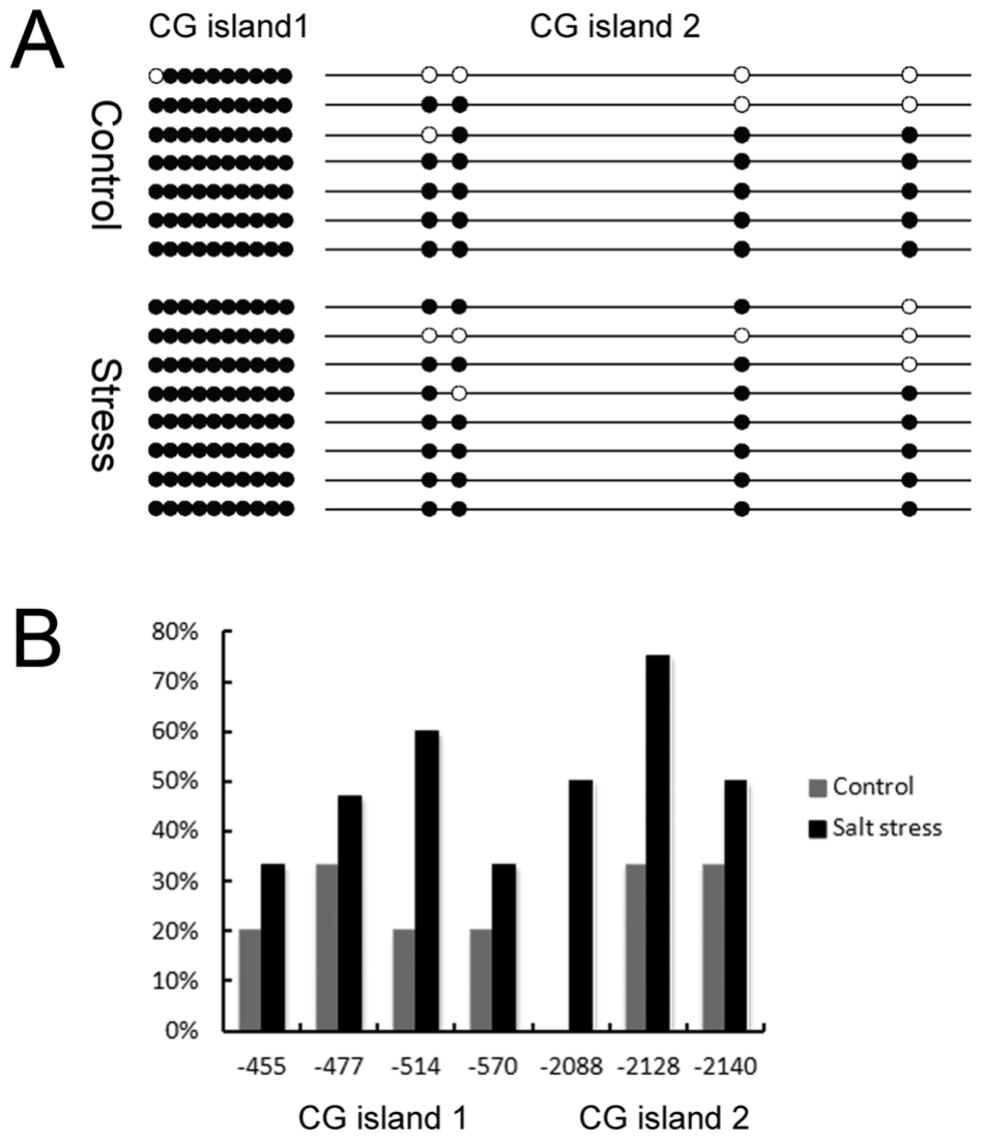
Methylation status of the *TaTIP2;2* promoter region as affected by salinity stress. (A) CG methylation (filled circles show methylated and open circles unmethylated CG sites). (B) CNG methylation. The horizontal axis shows the positions of CNG sites, and the vertical axis the proportion of methylated sites.

The 2.5kbp region upstream of the SR3 *TaTIP2;2* start codon contained two major CG islands, one located at positions -425 to -616, and the other at -1982 to -2,272. Bisulphate PCR analysis showed that almost all the cytosines present at CG dinucleotide sites were hyper-methylated irrespective of the presence of salinity stress (Figure 2A), while the methylation intensity at the CNG trinucleotide sites was increased by the imposition of salinity stress (Figure 2B).

### Sub-cellular distribution of TaTIP2;2 protein

**Figure 3:**
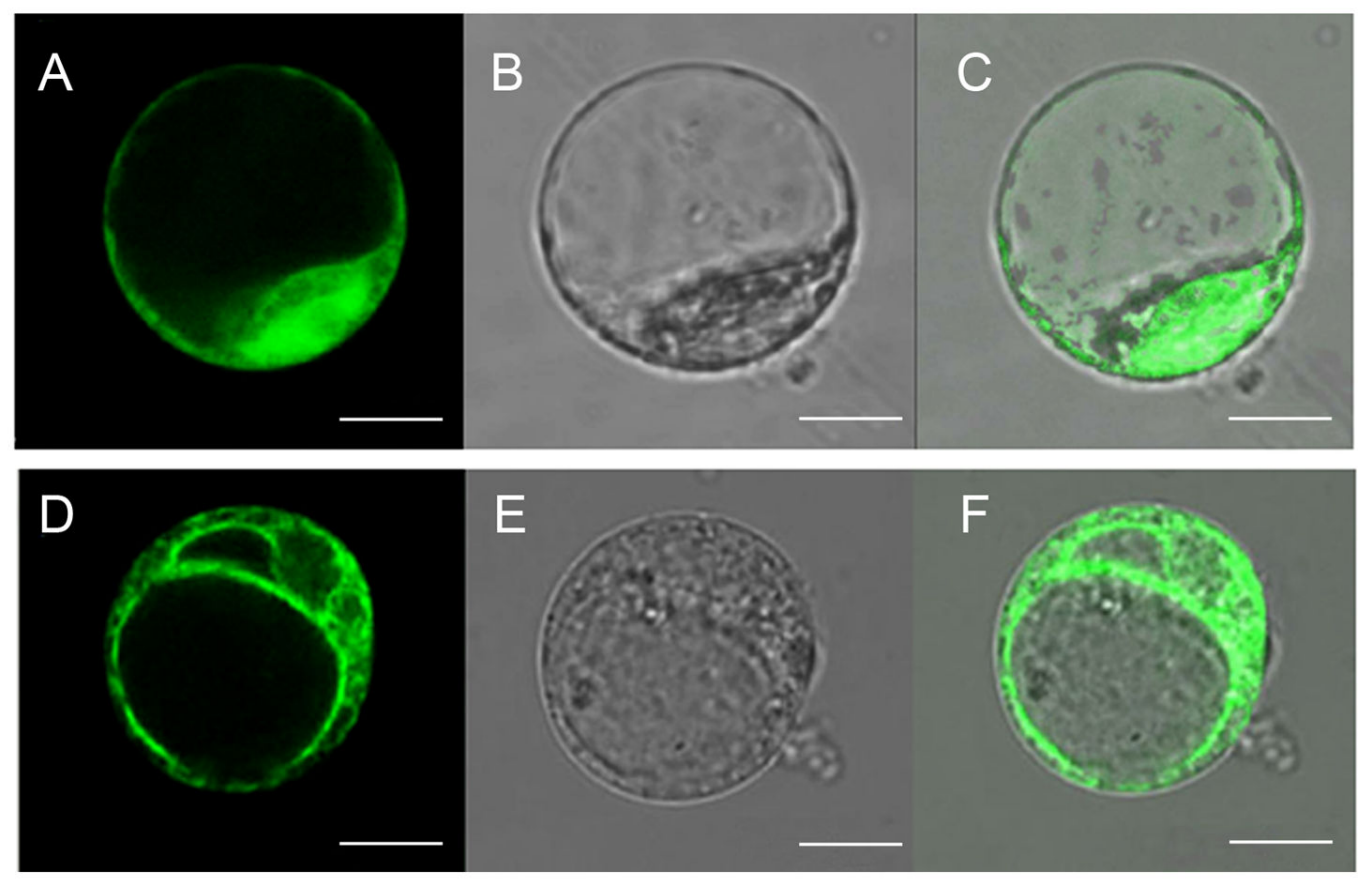
Sub-cellular localization of TaTIP2;2 protein in wheat protoplasts. (A-C) A transgene encoding GFP alone generates signal throughout the protoplast. (A) Fluorescent image, (B) bright field image, (C) merger of A and B. (D-F) The TaTIP2;2-GFP fusion is deposited in the endomembrane system. (D) Fluorescent image, (E) bright field image, (F) merger of D and E. Bars, 10μM.

Both GFP on its own and the TaTIP2;2::GFP fusion protein were transiently expressed in wheat protoplasts. In the case of the construct containing only *GFP*, confocal microscopy identified signal throughout the cytoplasm and nucleus (Figure 3A-C). However, in the case of the *TaTIP2;2::GFP* construct, there was no fluorescence on the cytoplasm membrane; rather the signal was concentrated in the tonoplast of the central vacuole and throughout the endomembrane system (Figure 3D-F).

### Heterologous expression of *TaTIP2;2* in stressed *A. thaliana*

**Figure 4:**
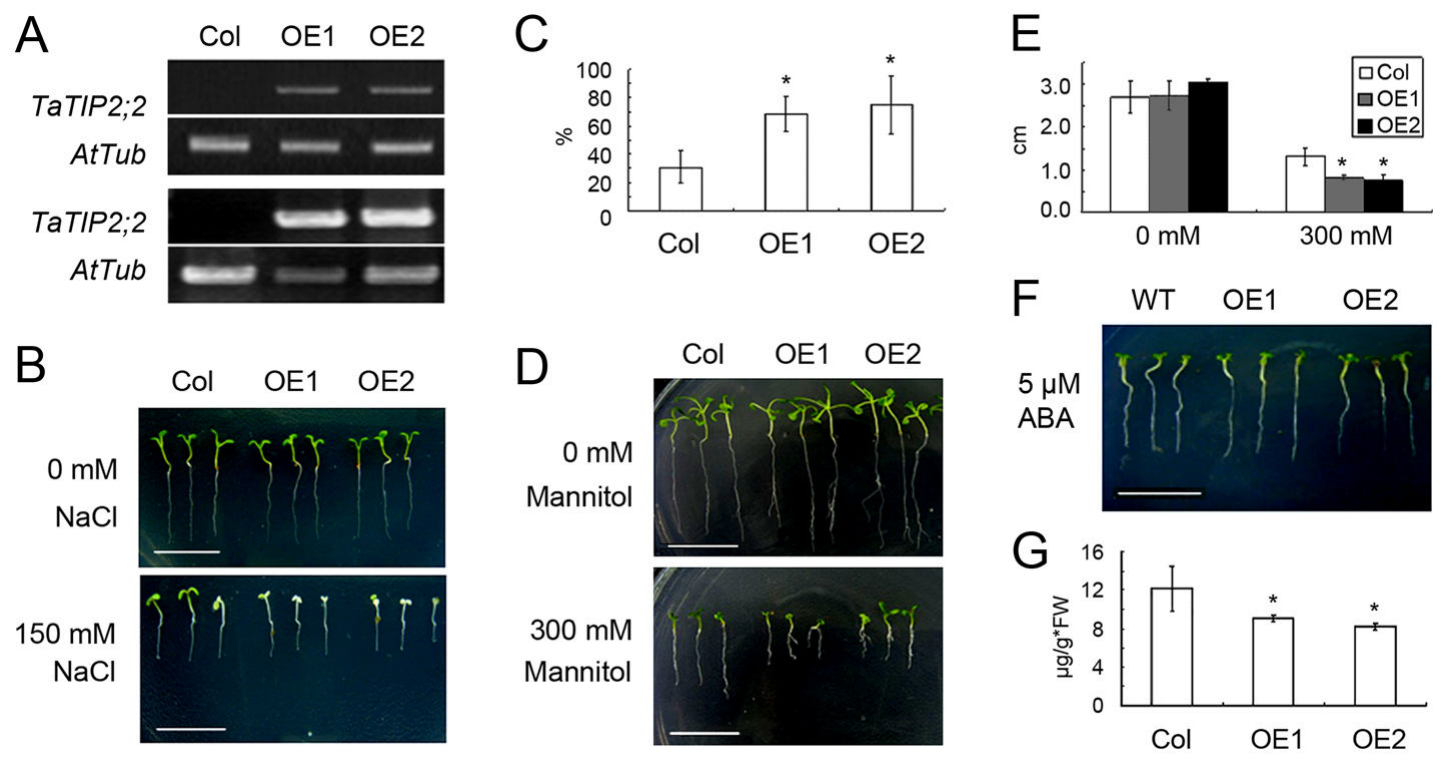
The behaviour of transgenic *A. thaliana* expressing ***TaTIP2;2***. (A) Genomic PCR (upper panel) and RT-PCR (lower panel) analysis shows that the T_3_ selections OE1 and OE2 carry and express the transgene. (B) The growth of wild type and transgenic plants challenged with salinity. (C) The rate of bleached progeny from wild type and transgenic plants exposed to 150mM NaCl. (D) The growth of wild type and transgenic plants challenged with mannitol. (E) Root growth of wild type and transgenic plants challenged with mannitol. (F) The growth of wild type and transgenic plants challenged with ABA. (G) The proline content of wild type and transgenic *A. thaliana*. Col: wild type Col-0 ecotype. Standard deviation was calculated with STDEVP function of Microsoft Excel 2010. Asterisks indicate significant differences between means (Student’s *t*-test, P<0.05).

Two homozygous transgenic T_3_ *A. thaliana* selections (OE1 and OE2) carried *TaTIP2;2* driven by the CaMV 35S promoter, and successfully expressed the gene (Figure 4A). The behaviour of OE1 and OE2 differed from that of the wild type control in response to exposure to ten days of salinity or osmotic (mannitol) stress. In the presence of NaCl, the growth of the transgenic plants ceased before the cotyledons had fully opened, and most of the seedlings were bleached; in contrast, the cotyledons of the wild type plants opened normally, and bleaching was less frequent (Figure 4B, C). The effect of mannitol stress was to shorten the length of the transgenics' primary roots compared to those of the wild type (Figure 4D, E). Thus the constitutive expression of *TaTIP2;2* in *A. thaliana* compromised the level of drought and salinity. There was no differential response to the supply of 5μM ABA (Figure 4F). The proline content in OE1 and OE2 was lower than in the wild type (Figure 4G).

### Effect of *TaTIP2;2* heterologous expression on the expression of abiotic stress-related genes

**Figure 5:**
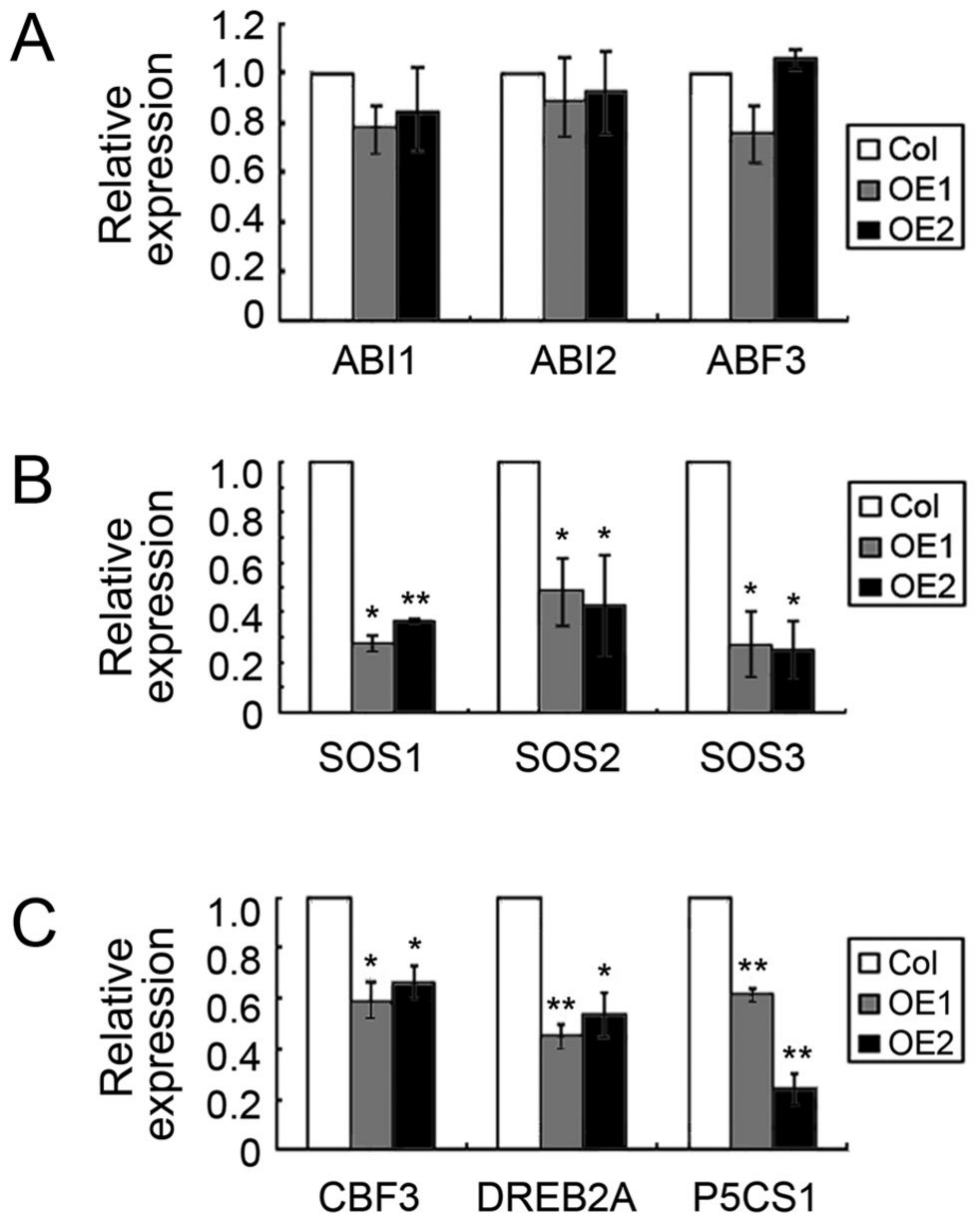
The effect of the heterologous expression of *TaTIP2;2* on the expression of abiotic stress-related genes in *A. thaliana*. (A) *ABI1*, *ABI2* and *ABF3* expression was not affected. (B) *SOS1*, *SOS2* and *SOS3* were down-regulated. (C) *CBF3, DREB2A* and *P5CS1* were down-regulated. Col: wild type Col-0 ecotype, OE1 and OE2: T_3_ selections expressing *TaTIP2;2*. Standard deviation was calculated with STDEVP function of Microsoft Excel 2010. Asterisks indicate significant differences between means (Student’s *t*-test, P<0.05 or 0.01).

As revealed by quantitative RT-PCR, the expression level of the ABA signalling genes *ABI1*, *ABI2* and *ABF3* was not obviously altered by the presence of the *TaTIP2;2* transgene (Figure 5A), similar results were also produced from *DREB2B*, *MYB2*, *MYC2*, *RAB18* and *RD29B* (data not shown), but that of *SOS1*, *SOS2*, *SOS3*, *CBF3*, *DREB2A* and *P5CS1* was markedly lowered (Figure 5B,C). 

## Discussion

### The participation of *TaTIP2;2* in the determination of stress tolerance

Response of plant to drought and osmotic stress is intimately related with water transport, in which the aquaporins are involved [8]. Although a number of attempts have been made to characterize the expression profile of various aquaporin genes [3,37], their *in vivo* function has been much less well researched [7,10,19,38]. Only few examples have been presented which suggest a role for them in stress response. In wheat, Gao et al. [19] showed that *TaNIP* activity can promote stress tolerance by increasing the K^+^/Na^+^ ratio via the regulation of the SOS pathway; wheat *PIP* aquaporins genes *TaAQP7* [21] and *TaAQP8* [20], durum wheat *PIP* genes *TdPIP1;1* and *TdPIP2;1* [22], cotton *PIP* gene *GhPIP2;7* [9] were also found to be beneficial to stress tolerance. However, many instances of variation in TIP aquaporin expression induced by abiotic stress have been documented. Thus, the down-regulation of both *ZmTIP2;2* and *TaTIP2;2* was induced by salinity [15,26,39] and that of *OsTaTIP2;2* by low temperature [17]. Here, we have demonstrated that the response of *TaTIP2;2* to the imposition of stress suggests that it acts as a negative regulator of salinity and drought stress. The observation that this response is independent of ABA is consistent with previous indications that *TIP* genes generally are not subject to hormonal regulation [14].

Gene expression can be regulated in a number of ways, but a prominent mechanism is represented by promoter methylation. We have shown that the *TaTIP2;2* upstream sequence experienced an increase in C-methylation at CNG sites as a result of the imposition of salinity stress, and that *TaTIP2;2* expression was reduced by this treatment. With respect to the regulation of gene expression, C-methylation at CNG sites is thought to be more important than at CG sites [40]. As a result, it is possible that these two events are causally related.

It has been suggested that the TIPs are deposited in the tonoplast. With the *A. thaliana* TIPs, AtTIP1;1 and AtTIP2;2 localized to the tonoplast of the root central vacuole and vacuolar bulbs [41]. Through an analysis of signal peptides or anchors, the site of TaTIP2;2 deposition has been bioinformatically predicted to lie within the plasma membrane [23], a conclusion inconsistent with the present experimental results, which showed that the protein was present not only in wheat protoplast tonoplasts, but also distributed throughout the endomembrane system (Figure 3D). The *P. ginseng* protein PgTIP1 appears to enhance the level of stress tolerance, but its sub-cellular location has not been determined [7]. A tomato TIP protein SlTIP2;2 was found localized to the tonoplast, and overexpression of it has increased stress tolerance [8]. Contradictory to this, *TaTIP2;2* has a negative effect on stress response. Potentially the localization of a given TIP may be related to its effect on the stress response, but as yet there are insufficient relevant data available to reach any sensible conclusion on this issue.

### *TaTIP2;2* is a negative regulator of stress tolerance

Despite the aquaporins (including the TIPs) having been the focus of a substantial body of research, there is little understanding of how they contribute to the stress response. The *A. thaliana* proteins AtTIP1;1 and AtTIP1;2 have been characterized as hydrogen peroxide channels, suggesting their possible role in the signalling of stress induced by reactive oxygen species [42]. Salinity stressed *A. thaliana* lines heterologously expressing *PgTIP1;1* can accumulate more Na^+^ and are more drought tolerant than the wild type [7]. SlTIP2;2 maintains the osmotic water permeability of tonoplast and extends the capacity of vacuole for osmotic buffering under stress [8]. Evidence for the involvement of TIPs in stress response signalling remains scanty.

Proline is frequently used by plants as an osmolyte, and its accumulation is a common response to a wide range of abiotic stresses [43,44]. Glutamate semialdehyde, the precursor of proline, is formed by the reduction of glutamate catalysed by pyrroline-5-carboxylate synthetase (P5CS) [43]. However, although the expression of *TaNIP* was found to be responsible for increasing the proline content of transgenic *A. thaliana*, it had no effect on the transcription of *P5CS1* [17]. In the present experiments, in contrast, the heterologous expression of *TaTIP2;2* did reduce *P5CS1* expression (Figure 5C), which may explain the lower proline content of the transgenic lines (Figure 4G). The implication is that *TaTIP2;2* expression reduced the osmotic tolerance of transgenic *A. thaliana* partially via the suppression of proline synthesis.

The SOS pathway genes *SOS1*, *SOS2* and *SOS3* are all positive regulators of salinity tolerance in *A. thaliana* [45]. SOS3 [46] is a calcium sensor, which activates the kinase activity of SOS2 [47]. The resulting complex up-regulates activity of SOS1 [48–50], a Na^+^/H^+^ antiporter located on the cell membrane that exports Na^+^ to the extracellular space and reduces the severity of the salinity stress [48,50]. Heterologous expression of *TaNIP* has been shown to reduce the level of *SOS2* expression, although surprisingly, the phenotypic effect of this reduction was to enhance abiotic stress resistance [19]. All three SOS genes were down-regulated by the *TaTIP2;2* transgene (Figure 5B), leading to our hypothesis that the negative impact of *TaTIP2;2* expression on the salinity tolerance of transgenic *A. thaliana* operates via its regulation of the SOS pathway.

Both *CBF3* [51] and *DREB2A* [52] are positive stress regulators acting independently of ABA. Their up-regulation has been shown to enhance the level of drought and salinity stress tolerance. Here, the heterologous expression of *TaTIP2;2* down-regulated both genes (Figure 5C), the effect of which would be expected to inhibit plant growth in the presence of abiotic stress. ABA plays a central role in determining stress tolerance [53]. The products of *ABI1* [54] and *ABI2* [55] are both negative regulators of ABA signalling, while that of *ABF3* [56] is a positive regulator. None of these three genes was transcriptionally affected by the heterologous expression of *TaTIP2;2* (Figure 5A). Expression of other tested ABA responsive stress regulators *MYB2*, *MYC2* [57,58], *RAB18* [59,60] and *RD29B* [58] was also not changed by the overexpression of *TaTIP2;2* (data not shown). These results suggest that the altered stress tolerance of the transgenic *A. thaliana* plants was not induced through an ABA-dependent pathway. Together with the response of *TaTIP2;2* in ABA treated wheat is not obvious (Figure 1D), it can be concluded that *TaTIP2;2* is ABA-independent. In the study of *A. thaliana* [13], rice [61], *Brassica napus* [12], *Craterostigma plantagineum* [62] and radish [14] aquaporin genes, ABA-dependent and independent members were both found, suggests that ABA-independent mechanism is commonly involved in the function of aquaporins.

As a channel protein gene, *TaTIP2;2* is behooved to lie at the most downstream of the signalling pathways. How its overexpression regulated several upstream genes of these signalling pathways still needs to be revealed. Our results show that TaTIP2;2 localizes in the endomembrane system (Figure 3). Given that tonoplast aquaporins are important for maintaining the osmotic equilibration in the cytoplasm [63], it could be suggested that overexpression of *TaTIP2;2* change the water balance inside the cells, and therefore lead to the change of the osmotic pressure in some compartments. This could be detected by osmosensors [57,58,64], which then triggers the signalling pathways and change the expression of stress responsive genes. On the other hand, auqaporins could transport solutes together with water, like H_2_O_2_, ammonia, glycerol, and so on [42,65–67]. Some of the solutes (for example, H_2_O_2_) are secondary messengers for stress response[68], which could also activate stress signalling and change the expression of the related genes.

In summary, it appears that *TaTIP2;2* acts as a negative regulator of the abiotic stress response. It is down-regulated by salinity-induced C-methylation in its promoter region. A possible scenario for improving the stress tolerance of wheat and other crops could be to decrease its expression using a genetic engineering approach.

## Supporting Information

Figure S1 **Phylogeny of plant TIP proteins.** AtTIPs from *A. thaliana*, HvTIPs from barley, OsTIPs from rice, TaTIPs from wheat, ZmTIPs from maize. TaTIP2;2 shown in bold type.
(TIF)
