# Supplementary figures and images for: Heterologous Expression of the Wheat Aquaporin Gene TaTIP2;2 Compromises the Abiotic Stress Tolerance of Arabidopsis thaliana
Source: PLoS One. 2013 Nov 4;8(11):e79618. doi: 10.1371/journal.pone.0079618 (PMC3817133; doi:10.1371/journal.pone.0079618)

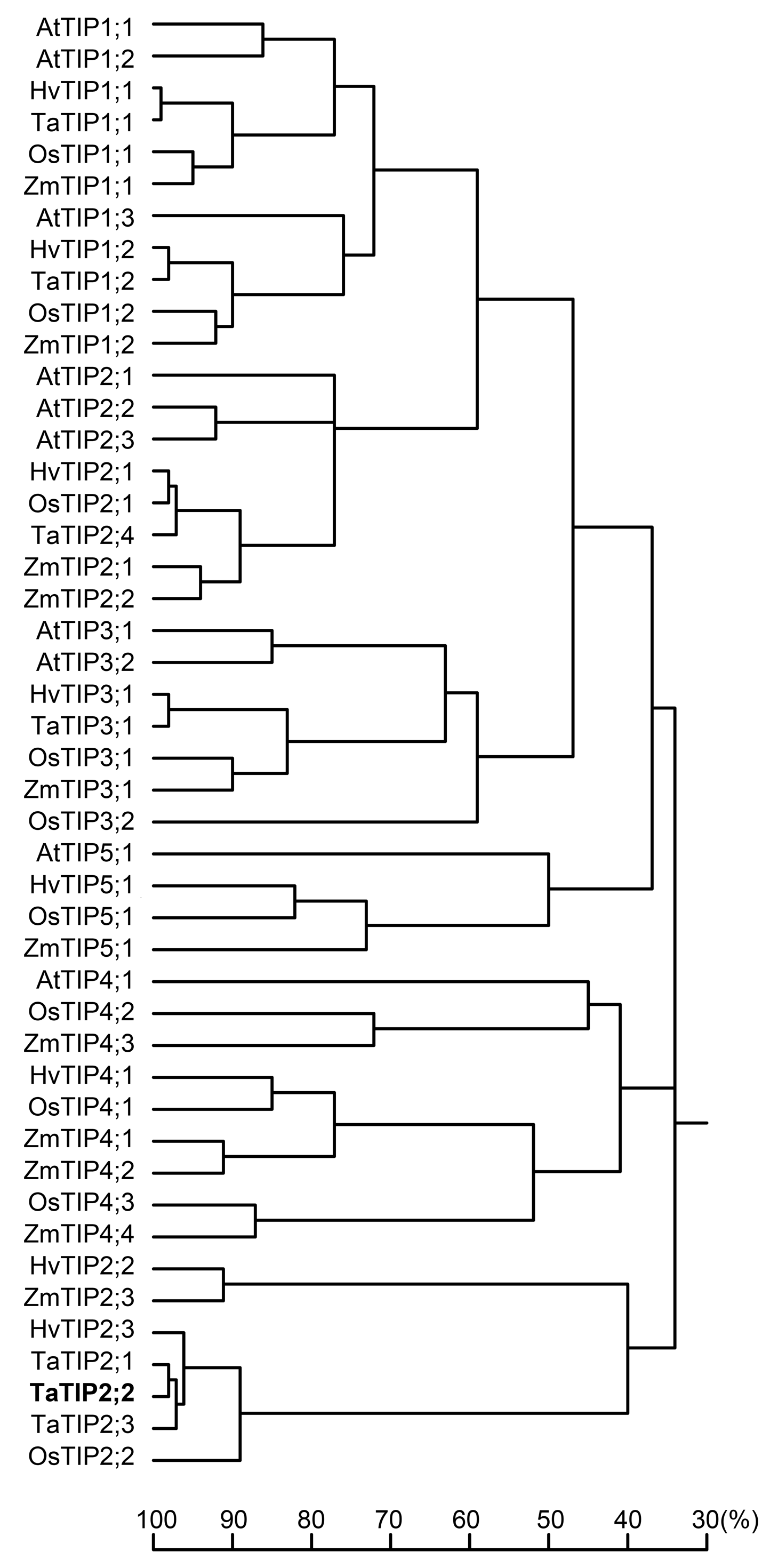

Supplement: Figure S1 — Phylogeny of plant TIP proteins. AtTIPs from A. thaliana, HvTIPs from barley, OsTIPs from rice, TaTIPs from wheat, ZmTIPs from maize. TaTIP2;2 shown in bold type. (TIF) [file pone.0079618.s001.tif]
